# Size‐Independent Transmembrane Transporting of Single Tetrahedral DNA Nanostructures

**DOI:** 10.1002/gch2.201900075

**Published:** 2019-11-20

**Authors:** Xi Chen, Falin Tian, Min Li, Haijiao Xu, Mingjun Cai, Qian Li, Xiaolei Zuo, Hongda Wang, Xinghua Shi, Chunhai Fan, Huricha Baigude, Yuping Shan

**Affiliations:** ^1^ School of Chemistry and Life Science Advanced Institute of Materials Science Changchun University of Technology Changchun Jilin 130012 China; ^2^ School of Chemistry & Chemical Engineering Inner Mongolia Key Laboratory of Mongolian Medicinal Chemistry Inner Mongolia University Hohhot Inner Mongolia 010020 China; ^3^ State Key Laboratory of Electroanalytical Chemistry Changchun Institute of Applied Chemistry Chinese Academy of Sciences Changchun Jilin 130022 China; ^4^ Laboratory of Theoretical and Computational Nanoscience CAS Key Laboratory for Nanosystem and Hierarchy Fabrication CAS Center for Excellence in Nanoscience National Center for Nanoscience and Technology Chinese Academy of Sciences Beijing 100190 China; ^5^ School of Chemistry and Chemical Engineering and Institute of Molecular Medicine Renji Hospital School of Medicine Shanghai Jiao Tong University Shanghai 200240 China

**Keywords:** force tracing, real‐time tracking, single tetrahedral DNA nanostructures, transmembrane kinetics

## Abstract

DNA nanostructures have attracted considerable attention as drug delivery carriers. However, the transmembrane kinetics of DNA nanostructures remains less explored. Herein, the dynamic process of transporting single tetrahedral DNA nanostructures (TDNs) is monitored in real time using a force‐tracing technique based on atomic force microscopy. The results show that transporting single TDNs into living HeLa cells need ≈53 pN force and ≈25 ms duration with the average speed of ≈0.6 µm s^−1^. Interestingly, the dynamic parameters are irrelevant to the size of TDNs, while the larger TDNs rotated slightly during the transporting process. Meanwhile, both the results from single‐molecule force tracing and ensemble fluorescence imaging demonstrate that the different size TDNs transmembrane transporting depends on caveolin‐mediated endocytosis.

## Graphical abstract

DNA nanostructures with inherent biocompatibility have become a unique vehicle in drug delivery. The transmembrane process is the first and the most important step for drug delivery. Herein, the transporting of single tetrahedral DNA nanostructures (TDNs) is recorded by a force‐tracing technique based on atomic force microscopy, and the size effect of TDNs on the transporting dynamics is studied.

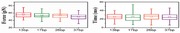

## 1 Introduction

Targeted drug delivery and precision medicine have now become a new paradigm in cancer therapy, with nanocarriers, pharmacologically active drugs could be directly delivered into target cancer cells to manage or reverse the course of disease. Nevertheless, the recent critical issue for drug delivery systems is lack of efficient drug delivery carriers.^1^, ^2^ DNA structures have gained much interest as a promising drug carriers, as a wide range of nanomaterials possess unique physical properties that can facilitate drug deliver system safer and more effective.^3^, ^4^, ^5^, ^6^ Several experiments proved that self‐assembled DNA nanostructures could enhance efficacies of chemotherapy, reduce adverse side‐effects, and even circumvent drug resistance.^7^

Among the DNA nanostructures, tetrahedral DNA nanostructures (TDNs), self‐assembling from four DNA strands with high yield, are considered as one of the simplest nanostructures.^2^, ^7^ These 3D TDNs are compact, mechanically stable, nontoxic, and resistant to nuclease degradation.^8^, ^9^ Fan's group found that as a multivalent drug carrier, the TDNs remained substantially intact after culturing in live cells for hours.^10^ Setyawati and co‐workers confirmed their superior efficacy on delivery anticancer drug.^11^ However, challenges remain in the employment of TDNS for cancer diagnostics and therapy, Recently, most reports focused on the transporting routes and the final destinations of TDNs in cells. The dynamic mechanism of TDNS entry into cells still unknown. Based on the theoretical simulation, Fan et al. proposed that the cellular internalization is the “like‐charge attraction.”^12^ However, theoretical simulation performed on bilayer model of phospholipids, the real situation of the TDNs transmembrane transporting on living cells is much more complicated. Further investigating the dynamic transmembrane mechanism of TDNs transporting into living cells with high temporal–spatial resolution are expected.

Herein, the dynamic transmembrane process of TDNs is tracked by force tracing technique based on atomic force microscopy (AFM), the force and duration of the transmembrane transport process of single nanoparticles/molecules could be detected down to pico‐newton and milliseconds, respectively.^13^, ^14^ Combining experiment and simulation, the TDNs size effect on the transmembrane dynamics and protein‐mediated mechanism was explored at single particle level under near physiological condition.

## 2 Results

### 2.1 Force Tracing Test of Single TDNs Transporting

**Figure 1:**
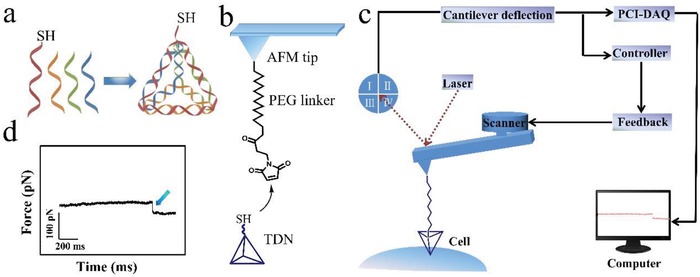
Schematic diagram of the force tracing test. a) Scheme of assembling the TDNs, from left to right, ssDNA are strands A, B, C, and D (red, orange, green, and blue), respectively. The strand A stretch out a 5‐base ssDNA strand with sulfydryl group. The strand C is labeled with cyanine‐3 (Cy3) fluorophores (Cy3‐TDNs). b) The diagram of connecting TDNs to the AFM tip, the TDNs is attached on the AFM tip with heterobifunctional PEG linker. c) The working principle of constant position mode force tracing technique. d) A typical force tracing curve with force signal (blue arrow).

HeLa cell line is selected as a model to study the dynamic transmembrane transporting process due to its higher activity of cellular uptake.^15^ The TDNs with different size synthesized by thermal cycler PTC‐200 were given as gift by Fan's group, TDN‐13, TDN‐17, TDN‐26, and TDN‐37, in which each edge of the TDN contains 13, 17, 26, and 37 base pairs, respectively (the preparation method and sequences of TDNs are shown in Table S1, Supporting Information).^16^ **Figure** **1**a shows the schematic diagram of TDNs assembling, the strand A was stretched out a 5‐basess DNA with sulfydryl group. The polyacrylamide gel electrophoresis analysis proved that the TDNs had been successfully synthesized (Figure S1, Supporting Information). To record the transmembrane process of single TDNs, it is essential to connect the TDNs to the AFM tip through heterobifunctional molecules, maleimide‐poly (ethylene glycol)‐succinimidyl carboxy methyl ester (MAL‐PEG2000‐SCM), as shown in Figure 1b. One side of the PEG linker was connected with the aminated AFM tip, and the sulfydryl group on TDNs reacted with the double bond moiety of the maleicamide through the addition reaction.^17^, ^18^ The successful attachment of TDNs on AFM tip was verified by fluorescence imaging (Figure S2, Supporting Information). The total length of heterobifunctional PEG linker and TDNs is 24–32 nm, which is long enough for testing the process of transmembrane (cell membrane thickness is about 20 nm).^19^, ^20^ The AFM tip tethered with TDNs could be located onto the living HeLa cell surface with the help of a CCD camera before performing force tracing test. After engaging the AFM tip on the cell membranes, the traditional force–distance curves were performed to find out the contact point between the TDNs attached AFM tip and the cell membrane (Figure S3, Supporting Information). Then the AFM tip was tardily moved to the contact point with lightly contacting the cell membrane through feedback system (details are shown in the Experimental Section). Figure 1c shows the schematic of constant position mode force tracing, where the laser beam is reflected by the AFM tip cantilever and recorded by the photodetector to detect the deflection change of the AFM tip cantilever. Once the TDN is internalized, the tethered AFM tip cantilever will bend downward, which will be recorded by a data acquisition card (PCI‐DAQ, with the sampling rate of 2 MS s^−1^, 2 million data points per second). To acquire clear force signal of the fast transporting process, the 20 kS s^−1^ sampling rate of data acquisition with a 100 low pass filter was applied. The transmembrane transporting of single TDN‐13 is first tracked, and the typical force tracing curve is shown in Figure 1d. The force tracing curve starts from the left to right, and the abscissa (X axis) presents the time, and the ordinate (Y axis) shows the deflection change of the AFM tip cantilever during the force tracing test that could be converted to the transporting force. The left side of the force curve is level, meaning the TDNs tethered AFM tip is just rested on the cell surface. While, cellular uptake of TDNs will make AFM tip cantilever bend downward, inducing an obvious force signal (blue arrow). After the internalization of TDNs, piezoelectric ceramics would drive the AFM tip cantilever to keep a force balance position, and the force tracing curve becomes level again. Due to the early stage features of the cellular uptake activity, the transmembrane transporting occasionally could not happen.^21^ Thousands of force tracing curves was collected on different cells of different positions to obtain hundreds of force tracing curves with force signal. To confirm the specificity of the force signal resulting from transporting events, control experiments were also performed. The clean AFM tips (without modification) and only PEG linker functionalized AFM tips were used to perform force tracing curves, respectively, and there was no force signal observed in thousands of force tracing curves (Figure S4, Supporting Information).

### 2.2 Size‐Independent of TDNs Transmembrane

**Figure 2:**
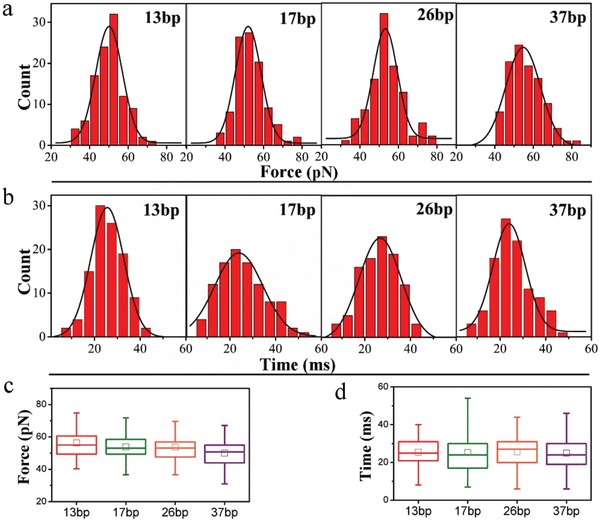
The transporting force and duration of single TDNs with different size. The histograms of transporting force a) and duration b) for TDN‐13, TDN‐17, TDN‐26, and TDN‐37. The box chart statistical comparison of transporting force c) and duration d). The vertical lines through boxes represent the distribution of values, the boxes mean the main distribution (50%) of values, the squares in every box represent the average value, and the horizontal lines in each box show the median. (*n* ≈ 100)

To explore the size effect, transmembrane transporting measurements of TDNs with different size (TDN‐13, TDN‐17, TDN‐26, and TDN‐37) were performed, respectively, and the typical force tracing curves are similar. Interestingly, it is found that both the transporting force and duration were undifferentiated. As shown in **Figure** **2**, the transporting force ranges from 30 to 95 pN, and the average value for TDN‐13, TDN‐17, TDN‐26, and TDN‐37 is 49.9 ± 7.9, 53.6 ± 8.7, 53.8 ± 8.9, and 56.2 ± 8.7 pN, respectively (Figure 2a). Figure 2b shows the corresponding duration, which ranges from 5 to 50 ms with the mean value of 25.4 ± 6.8, 25.3 ± 11.3, 25.6 ± 8.1, and 24.9 ± 8.4 ms for TDN‐13, TDN‐17, TDN‐26, and TDN‐37, respectively. The further box chart statistical comparison is shown in Figure 2c,d. It is noted that as the size of TDNs increasing, both the force and duration for the transmembrane transporting activity remain essentially the same. These results indicate that the transmembrane force and duration of TDNs are size‐independent, which is different from that of spherical nanoparticles and other anisotropic ones.^22^, ^23^

What about other dynamic parameters of TDNs in transmembrane transporting? the TDNs engulfment depth *D* was calculated, which includes the bending distance of the AFM cantilever tip (*d*) and the extension length of the PEG linker (*h*)

(1) D = h + d

Using the extended worm‐like chain model, the stretched length of the PEG linker could be calculated, which characterizes the force (*F*)‐dependent stretching activity by the following equation^13^

(2) $$\frac{FL_{P}}{k_{B}T}\;=\;\frac{1}{4}{\left(1\;-\;\frac{h}{L_{0}}\;+\;\frac{F}{K_{0}}\right)}^{-2}-\frac{1}{4}\;+\;\frac{h}{L_{0}}\;-\;\frac{F}{K_{0}}$$

where *k*_B_ is the Boltzmann constant, *T* is the absolute temperature, *L*_p_ is the persistence length, *K*_0_ is the enthalpic correction, *h* is the extension of PEG linker, and *L*_0_ is the contour length. Based on the previous literature,^13^ the persistence length *L*_p_ is 3.8 ± 0.02 Å, and the enthalpic correction *K*_0_ is 1561 ± 33 pN. Given that the PEG unit length is 4.2 Å, the estimated contour length *L*_0_ to PEG linker is about 196 Å. Based on Equation (2), *h* is calculated as 14.28, 14.43, 14.49, and 14.59 nm for TDN‐13, TDN‐17, TDN‐26, and TDN‐37, respectively. The bending distance of the AFM tip cantilever is calculated using the following Hooke's law^24^

(3) F=K× d

where *F* represents the force of single TDNs transmembrane transporting on living HeLa cells that could be directly measured from the force tracing curves, and *K* is the spring constant of the AFM tip cantilever. According to Equations (1)–3), the engulfment depth *D* is calculated as 14.89, 15.08, 15.15, and 15.28 nm for TDN‐13, TDN‐17, TDN‐26, and TDN‐37, respectively (Figure S5, Supporting Information). Therefore, the average speed of single TDNs transmembrane transporting on living HeLa cells could be calculated as 0.586, 0.596, 0.592, and 0.613 µm s^−1^ for TDN‐13, TDN‐17, TDN‐26, and TDN‐37, respectively (engulfment depth *D* divided by duration, e.g., 14.89 nm/25.4 ms). These results further confirmed the statement that the single TDNs transporting on living cells is size‐independent.

### 2.3 Theoretical Simulation of TDNs Transporting

To further elucidate the size‐irrelevant transporting of TDNs, the dissipative particle dynamics (DPD) simulation of different size TDNs transporting was conducted based on a dipalmitoylphosphatidylcholine lipid bilayer model. When the length of DNA molecules is short, they are frequently treated as the rigid rods. Therefore, in the simulation, single TDNs is constructed by six rigid rods with the same length (Figure S6, Supporting Information). Similar to the lipid model developed by Sunil Kumar et al.,^25^, ^26^, ^27^ the lipid and receptor are represented by four spherical beads, as shown in Figure S6 (Supporting Information). The TDNs interacting with the receptors on cell membrane is simulated through a soft LJ potential (see Method in the Supporting Information). For the initial simulation, the TDNs are positioned in close proximity above the surface of a bilayer. Then the TDNs are pulled into the bilayer and wrapped.

**Figure 3:**
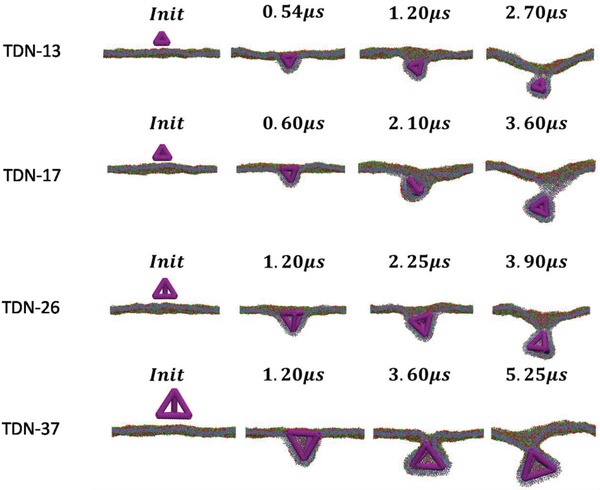
Time sequence of the snapshots for interactions between different size TDNs (purple)and membranes.

**Figure 4:**
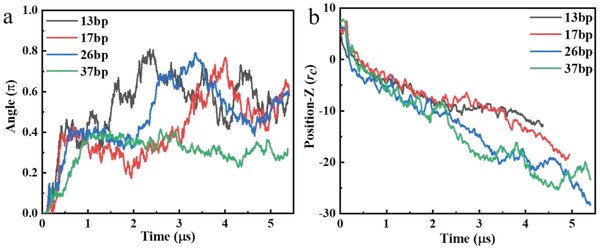
Simulation analysis. a) Time evolution of the angle for the TDNs with different size during simulation. b) Time evolution of the TDNs position relative to the middle line of the membrane.

**Figure** **3** shows the typical simulation snapshots of the TDNs‐membrane interaction. Initially, the TDNs were placed above the membrane surface with one of their facets faced up with the membrane. As the simulation continuing, the TDNs entered into the lipid membrane in a short time, and then a remarkable rotation with a value of about π/2 made the TDNs face the membrane with one of its corners due to the attraction between the TDNs and receptors (**Figure** **4**a). After that, the TDNs were wrapped and internalized as a“corner attack” like structure gradually. In addition, when three of the TDNs facets nearest the corner were wrapped fully, the TDNs would rotate continuously to complete the finally full wrapping. It is found that the wrapping time of the smallest TDN‐13 is a little bit shorter than that of the largest TDN‐37 (Figures 3 and 4b). However, the difference of wrapping time between the different TDNs is insignificant comparing to their difference of volume (volume of the TDN‐13 is 10 nm^3^, and TDN‐37 is 280 nm^3^). Therefore, the dynamic process of TDNs transmembrane transporting is size‐independent in the range of several tens of nanometers. Comparing the experimental and simulation results, we found that the transporting duration from experiments was longer attributed to the complexity of real cell membrane. The complicated cell membranes have been described as the protein layer–lipid–protein Island model that the proteins at the ectoplasmic side of the cell membrane form a dense protein layer showing a smooth feature with a height of about 4 nm.^19^, ^20^ Interestingly, the TDNs with different size showed different rotation angle in the final stage. The rotation of the smaller TDNs (TDN‐13, TDN‐17, and TDN‐26) seemed more active, with one of its corners contacted with the membrane when it entered into the bilayer. While the larger TDN‐37 rotated slightly and finally entered the bilayer (Figure 4).

### 2.4 Protein‐Mediated Mechanism of TDNs Transmembrane

**Figure 5:**
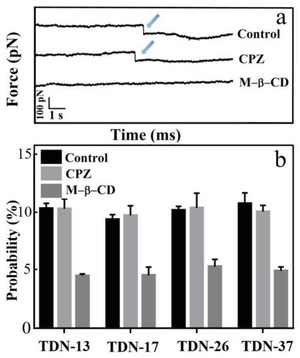
Block experiments. a)The typical force tracing curves of TDN‐13 modified AFM tips on the HeLa cells before (Control) and after inhibiting (CPZ, M‐β‐CD). b) The probability of force tracing curves with force signal for TDN‐13, TDN‐17, TDN‐26, and TDN‐37, respectively. Values represent mean ± standard deviation.

The transporting route of single TDNs with different size was also investigated. According to previous reports, clathrin‐dependent and caveolin‐dependent endocytotic processes are the two major pathways of endocytosis.^28^ Herein, the inhibitors of methyl‐β‐cyclodextrin (M‐β‐CD) and chlorpromazine (CPZ) were used to explore endocytic pathway of TDNs with different size, as shown in **Figure** **5**. After incubation the HeLa cells with CPZ (10 µg mL^−1^) for 30 min at 37 °C, the probability of force tracing curves with force signal is 10.3%, 9.7%, 10.4%, and 10.1% for TDN‐13, TDN‐17, TDN‐26, and TDN‐37, respectively. The probability is similar to that of untreated HeLa cells (Control, 10.3%, 9.4%, 10.2%, and 10.8% for TDN‐13, TDN‐17, TDN‐26, and TDN‐37, respectively). After inhibiting with M‐β‐CD (5 × 10^−3^ m) for 30 min at 37 °C, the probability of force tracing curves with force signal decreased to 4.5%, 4.6%, 5.3%, and 4.9% for TDN‐13, TDN‐17, TDN‐26, and TDN‐37, respectively (Figure 5b). It is demonstrated that caveolin is related to the endocytosis of single TDNs, which is consistent with Fan's report that TDN‐17 entry into cells depends on caveolin‐mediated endocytosis.^10^

**Figure 6:**
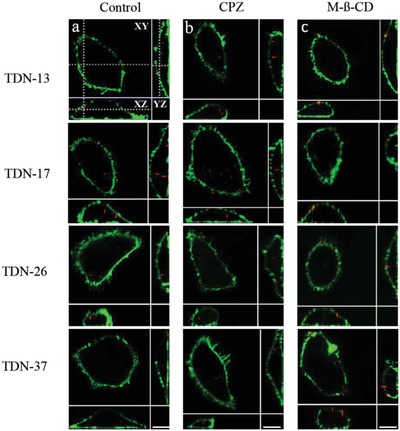
The fluorescence imaging of TDNs in HeLa cells with two‐color confocal microscopy. a) The fluorescence images of HeLa cells after coincubation with TDN‐13, TDN‐17, TDN‐26, and TDN‐37, respectively, the TDNs (red) are located inside the cell membranes (green). b,c) Fluorescence images of TDNs and HeLa cells after inhibiting with CPZ and M‐β‐CD, respectively. The cell membranes were stained with lipophilic dyes DIO (green), and the TDNs labeled by Cy3 (red), scale bar: 10 µm.

In order to further prove the transporting route, two‐color fluorescence imaging with confocal microscopy was performed. Before inhibiting, 3D fluorescence images of HeLa cells demonstrated the activity of TDNs entry into the cells (Control). The colocalization images of the YZ plane and XZ cross‐section confirmed that the TDNs with different sizes were located inside the cells, as shown in **Figure** **6**a. After pretreating the cells with inhibitor CPZ (10 µg mL^−1^, 30 min), the TDNs (red) still could be found inside the cells. However, after inhibition with M‐β‐CD (5 × 10^−3^ m, 30 min), almost no TDNs was found inside the cells (Figure 6). The results are consistent with that from force tracing technique, the main transmembrane transporting pathway of the TDNs is caveolin‐mediated endocytosis.

## 3 Discussion

DNA nanostructures with inherent biocompatibility and biodegradability become a unique vehicle in drug delivery.^29^, ^30^, ^31^ In the process of drug delivery, transmembrane is the first and the most important step. Nevertheless, the dynamic transmembrane mechanism of the DNA nanostructures remains poorly understood. The dynamic parameters (force, duration, and speed) for transporting single DNA nanostructures in livng cells have not been described. In biological and biomedical applications of DNA nanostructures, the varying dimensions, shapes, and geometries all affect during the transmembrane transporting process.^32^, ^33^ Based on the theoretical simulation, it is reported that TDNs could enter into the cells with corner by reducing the influence of electrostatic force, and the dynamic process may result in the charge redistribution of the mobile cell membranes.^10^, ^12^ However, due to the limitation of present computing abilities, the size of the modeled DNA structures is limited within ≈10 nm.

Herein, the force tracing technique with high temporal–spatial resolution was used to track the transmembrane dynamic process of transporting single TDNs into living cells, which has sucessfully monitored the dynamic transporting of exogenous materials from several nanometers to around 1 µm on living cell.^22^, ^24^, ^34^, ^35^ Based on the force tracing technique, the real‐time transmembrane kinetics of single TDNs with the size from 4.4 to 12.6 nm was monitored, they all need ≈53 pN force and ≈5 ms duration (Figure 2), as well as the similar displacement (Figure S5, Supporting Information) with the average speed of ≈0.6 µm s^−1^. Meanwhile, the Z‐position of the different size TDNs relative to the middle line of the membrane changes in similar trend as time going (Figure 4b), and the different size TDNs are fully wrapped at the same time during the cell entry (Figure S7, Supporting Information). The size‐independent transmembrane of TDNs could be attributed to the “like‐charge attraction” mechanism at the interface of cytoplasmic membranes, the TDNs primarily approach cells with corners to minimize electrostatic repulsion, inducing uneven charge redistribution in the membrane under the short‐distance confinement by caveolin. The corner attack of TDNs and the charge redistribution of the membrane play important role in the process of size‐independent transmembrane.^12^ The process is different from the size‐dependent uptake of some other nanoparticles.^23^ It is also worthwhile to note that the larger TDNs rotated slightly during the transmembrane process due to the limitation of the rotation freedom (Figure 4a). The reslut is consistent with the previous conclusion that during the cell entry of TDN‐20, the orientation adjustment plays the significant role, which implying that the difference in their rotation freedom possibly accounts for the difference in cell entry.^12^ The results from single particle force tracing and ensemble fluorescence imaing demostrated that the TDNs transmembrane depend on caveolin‐mediated endocytosis.

## 4 Conclusion

In the present study, the transmembrane transportation behavior of TDNs was monitored by using force tracing technique in real‐time at single particle level, and the dynamic parameters of transporting TDNs were measured. Both the simulation and experimental data established that the dynamic parameters of TDNs transporting were nearly size‐independent, including transporting force, duration, displacement, and average speed. Moreover, the TDNs with different size transporting depended on caveolin‐mediated endocytosis pathway. Taken together, this report will open new opportunities for investigated a wider spectrum of DNA stuctures as drug carriers, and sheds light on designing novel cellular delivery strategies.

## 5 Experimental Section

*Cell Culture*: HeLa cells were purchased from shanghai institute of biological science, which were cultured in Dulbecco's Modified Eagle Medium (DMEM) medium with 10% fetal bovine serum (FBS), 100 units mL^−1^ penicillin (Invitrogen), and 100 mg mL^−1^ streptomycin (Invitrogen) at 37 °C under the atmosphere 5% CO_2_. To remove cell debris and unattached cell, the cells were washed with phosphate buffer (PBS) for three times before performing all subsequent experiments.

*AFM Tip Modification*: Modification of AFM tips (PPP‐BSI) need two steps. AFM tips were washed by Piranha solution (V(H_2_SO_4_):V(H_2_O_2_) = 3:1) for 1 h, cleaned with ultrapure water twice and absolute ethylacohol once then the AFM tips were dried by argon gas, and cleaned under O_3_ for 30 min to remove other impurities. After cleaning, the AFM tips were modified with 3‐aminopropyltriethoxysilane^17^ to generate amino group, it was convenient for linking the heterobifunctional PEG (MAL‐PEG2000‐SCM, FW≈2000, SensoPathechnologies, Bozeman, MT 1 mg mL^−1^). After drying with argon, the tips were immersed in a mixture of 100 × 10^−9^ m TDNs, 50 µL NH_2_OH‐reagent (500 × 10^−3^ m NH_2_OH^•^HCl, 25 × 10^−3^ m EDTA, pH 7.5), and 50 µL buffer A (100 × 10^−3^ m NaCl, 50 × 10^−3^ m NaH_2_PO_4_, 1 × 10^−3^ m EDTA, pH 7.5). After functionalization for 1 h, the AFM tips were washed with PBS for three times and stored at 4 °C.

*Fluorescence Imaging of AFM Tips*: Fluorescence imaging of TDNs modified AFM tips was carried out with Andorra Q3.6.2 confocal microscopy. Cy3 labeled TDNs were excited with a 561 nm helium–neon laser. The fluorescence images were collected with a NA = 1.40, 100 × oil immersion objective. The fluorescence imaging was analyzed by Image J.

*Force Tracing Experiments*: As the sample of force tracing test, HeLa cells were seeded on glass slide in 35 mm petri dish for 1 or 2 d until 75% of the petri dish was covered by HeLa cells. Before performing force tracing experiments, the HeLa cells were washed with PBS for three times and then added to 2 mL DMEM without FBS. In block experiments, the cells were precultured with CPZ (ultimate concentration 10 µg mL^−1^) for 30 min and methyl‐β‐cyclodextrin (M‐β‐CD, ultimate concentration 10 × 10^−3^ m) for 30 min at 37 °C, respectively.

Force tracing curves were obtained with AFM 5500 (Agilent Technologies, Chandler, AZ), and controlling the temperature at 37 °C with temperature controller 325 (Agilent Technologies, Chandler, AZ). The sensitivity and the spring constant of AFM tip was set right as reported previously.^36^ After finding the contact point through force–distance curve, the AFM tip was gradually approached the contact point, while the proportional‐integral control system (*P* = 0.001; *I* = 0.001; the error signal between the set point and the deflection of the cantilever is −2.0 V) would be turned off with the TDNs tethered AFM tip lightly contacting cell surface. The transporting of TDNs that attached on the AFM tip will cause the deflection changes of AFM tip cantilever, which were recorded by a 16‐bit DA/AD card (PCI‐6361e, National Instruments) controlled by LabVIEW software, and the data were processed with MATLAB 7.9 software (Math Works Inc, Natick, MA).

*The Fluorescence Imaging of HeLa Cells and TDNs*: For fluorescence imaging, HeLa cells were incubated with TDNs (100 × 10^−9^ m) for 1 h at 37 °C, then washed by PBS for three times. Subsequently, the cell membranes were stained with lipophilic dyes DIO (1 mg mL^−1^) for 30 min at room temperature, washed by PBS for three times to remove extra dyes, and added 1 mL PBS to proceed fluorescence experiment. Finally, the cells were fixated with paraformaldehyde (4%), after washing with PBS. For block experiments, before incubation with TDNs, the HeLa cells were cultured with blocking reagents CPZ (ultimate concentration 10 µg mL^−1^) and methyl‐β‐cyclodextrin (M‐β‐CD, ultimate concentration 10 × 10^−3^ m) for 30 min, respectively. Fluorescence imaging was performed using Andorra Q3.6.2 confocal microscopy. Cy3 labeled TDNs were excited with a 561 nm helium–neon laser. The fluorescence images were collected using a NA = 1.40, 100 × oil immersion objective. In order to present a good visual effect, all images were added false colors. All fluorescence images were analyzed using Image J.

*Molecular Simulation*: DPD method was used to elucidate the transporting mechanism of TDNs with different size. To simplify the system, a model comprising a piece of cellular membrane and TDNs was constructed. The cellular membrane was formed by lipids and receptors that were a stable bilayer structure like Sunil Kumar et al. model.^26^, ^37^ The TDNs were formed by beads arranged as a tetrahedron and constrained to move as a rigid body during the simulation. In order to mimic the receptor–ligand interaction, a modified soft LJ potential was used during the TDN‐membrane interaction simulation. The bonded interaction between neighboring beads of the lipid, receptor, and ligand was described by a harmonic spring force. The stiffness of the lipid membrane was constrained by a three‐body bond angle potential. The interaction between each pair of DPD beads was governed by Newton's equation of motion d*v*_ij_/d*t* = F*_ij_*/m, and the total force F*_ij_* applied on each bead *i*, because that bead *j* was given as a sum of five terms, i.e., conservative force $\left(F_{ij}^{c}\right)$, dissipative force $\left(F_{ij}^{D}\right)$, random force $\left(F_{ij}^{R}\right)$, bond force $\left(F_{ij}^{B}\right)$ (including the harmonic bond force and bond angle force), and the LJ interaction between ligands and receptors. The details of the setup of the system and the interaction potential were listed in the simulation method.

The simulation box is a cube of size 80r_c_ x 80r_c_ x 60r_c_ with periodic boundary condition applied three directions. There are total 1 152 000 beads in the simulation box to keep the particle density. To avoid the distribution of the nanoplate to the equilibrium state of a pure membrane, a long time (≈300 ns) equilibrium simulation was preformed when the TDN was placed near the membrane surface. All simulations in this work were carried out by using the modified soft package Lammps.

## Conflict of Interest

The authors declare no conflict of interest.

## Supporting information

Supporting Information
