# Supplementary material for: Size‐Independent Transmembrane Transporting of Single Tetrahedral DNA Nanostructures
Source: Glob Chall. 2019 Nov 20;4(3):1900075. doi: 10.1002/gch2.201900075 (PMC7050086; doi:10.1002/gch2.201900075)
Supplement: Supplementary file 1 — Supporting Information [file GCH2-4-1900075-s001.pdf]

Copyright WILEY-VCH Verlag GmbH & Co. KGaA, 69469 Weinheim, Germany, 2019.

Global Challenges
Open Access

Supporting Information

for *Global Challenges*, DOI: 10.1002/gch2.201900075

Size-Independent Transmembrane Transporting of Single Tetrahedral DNA Nanostructures

Xi Chen, Falin Tian, Min Li, Haijiao Xu, Mingjun Cai, Qian Li, Xiaolei Zuo, Hongda Wang, Xinghua Shi, Chunhai Fan,* Huricha Baigude,* and Yuping Shan**

Supporting Information

Size-Independent Trans-membrane Transporting of Single Tetrahedral DNA Nanostructures

Xi Chen, Falin Tian, Min Li, Haijiao Xu, Mingjun Cai, Qian Li, Xiaolei Zuo, Hongda Wang, Xinghua Shi, Chunhai Fan*, Huricha Baigude*, Yuping Shan**

Preparation of TDNs

Tetrahedral DNA nanostructures (TDNs) were synthesized by mixing equimolar (1 μM) oligonucleotides (A, B, C, and D) in TM buffer (20 mM Tris, 50 mM $MgCl_2$, pH 8.0). The solution was heated to 90 °C and kept for 10 min, then cooled down to 4 °C rapidly, and lasted for at least 20 min in a thermal cycler PTC-200 (MJ. Research Inc, SA). All TDNs were characterized by 8% polyacrylamide gel electrophoresis (1 × TBE containing 12.5 mM $Mg(AC)_2$) and imaged by a chemiluminescence imaging system (G: BoxChemi-XL) after staining with 0.01% gel red.[1]

Table S1 DNA sequences used in the experiments.

	DNA	Sequences(5'-3')
TDN-13	A13-5T-SH	TTTTTACACTACGTCAGAACAGCTTGCATCACTGGTCACCAGAGTA
	B13	ACGAGCGAGTTGATGTGATGCAAGCTGAATGCGAGGGTCCT
	C13-Cy3	TCAACTCGCTCGTAACTACACTGTGCAATACTCTGGTGACC
	D13	TCTGACGTAGTGTATGCACAGTGTAGTAAGGACCCTCGCAT
TDN-17	A17-5T-SH	TTTTTACATTCCTAAGTCTGAAACATTACAGCTTGCTACACGAGAAGAGCCGCCATAGTA
	B17	TATCACCAGGCAGTTGACAGTGTAGCAAGCTGTAATAGATGCGAGGGTCCAATAC
	C17-Cy3	TCAACTGCCTGGTGATAAAACGACACTACGTGGGAATCTACTATGGCGGCTCTTC
	D17	TTCAGACTTAGGAATGTGCTTCCCACGTAGTGTCGTTTGTATTGGACCCTCGCAT
TDN-26	A26-5T-SH	TTTTTGCCTGGAGATACATGCACATTACGGCTTTCCCTATTAGAAGGTCTCAGGTGCGCGTTTCGGTAAGTAGACGGGACCAGTTCGCC
	B26	CGCGCACCTGAGACCTTCTAATAGGGTTTGCGACAGTCGTTCAACTAGAATGCCCTTTGGGCTGTTCCGGGTGTGGCTCGTCGG
	C26-Cy3	GGCCGAGGACTCCTGCTCCGCTGCGGTTTGGCGAACTGGTCCCGTCTACTTACCGTTTCCGACGAGCCACACCCGGAACAGCCC

	D26	GCCGTAATGTGCATGTATCTCCAGGCTTTCCGCAGCGGAGCAGGAGTCCTCGGCCTTTGGGCATTCTAGTTGAACGACTGTCGC
TDN-37	A37-1	CCCTGTACTGGCTAGGAATTCACGTTTTAATCTGGGCTTTGGGTTAAGAAACTCCCCG
	A37-2	CGCTGGAGGCGCATCACCGTTTGCGTATGTGTTCTGTGCGGCCTGCCGTCCCGTGTGGG
	B37-1	CGGTGATGCGCCTCCAGCGCGGGGAGTTTCTTAACCCTTTCCGACTTACAAGAGCCGG
	B37-2	GCGAGACTCAGGTGGTGCCTTTGGCATTCGACCAGGAGATATCGCGTTCAGCTATGCCC
	C37-1-Cy3	CCCATGAGAATAATACCGCCGATTTACGTCAGTCCGGTTTCCCACACGGGACGGCAGGC
	C37-2	CGCACAGAACACATACGCTTTGGGCATAGCTGAACGCGATATCTCCTGGTCGAATGCC
	D37-1-5T-SH	TTTTTGCCCAGATTAAAACGTGAATTCCTAGCCAGTACAGGGTTTCCGGACTGACGTAAATCGG
	D37-2	CGGTATTATTCTCATGGGTTTGGCACCACCTGAGTCTCGCCCGGCTCTTGTAAGTCGG

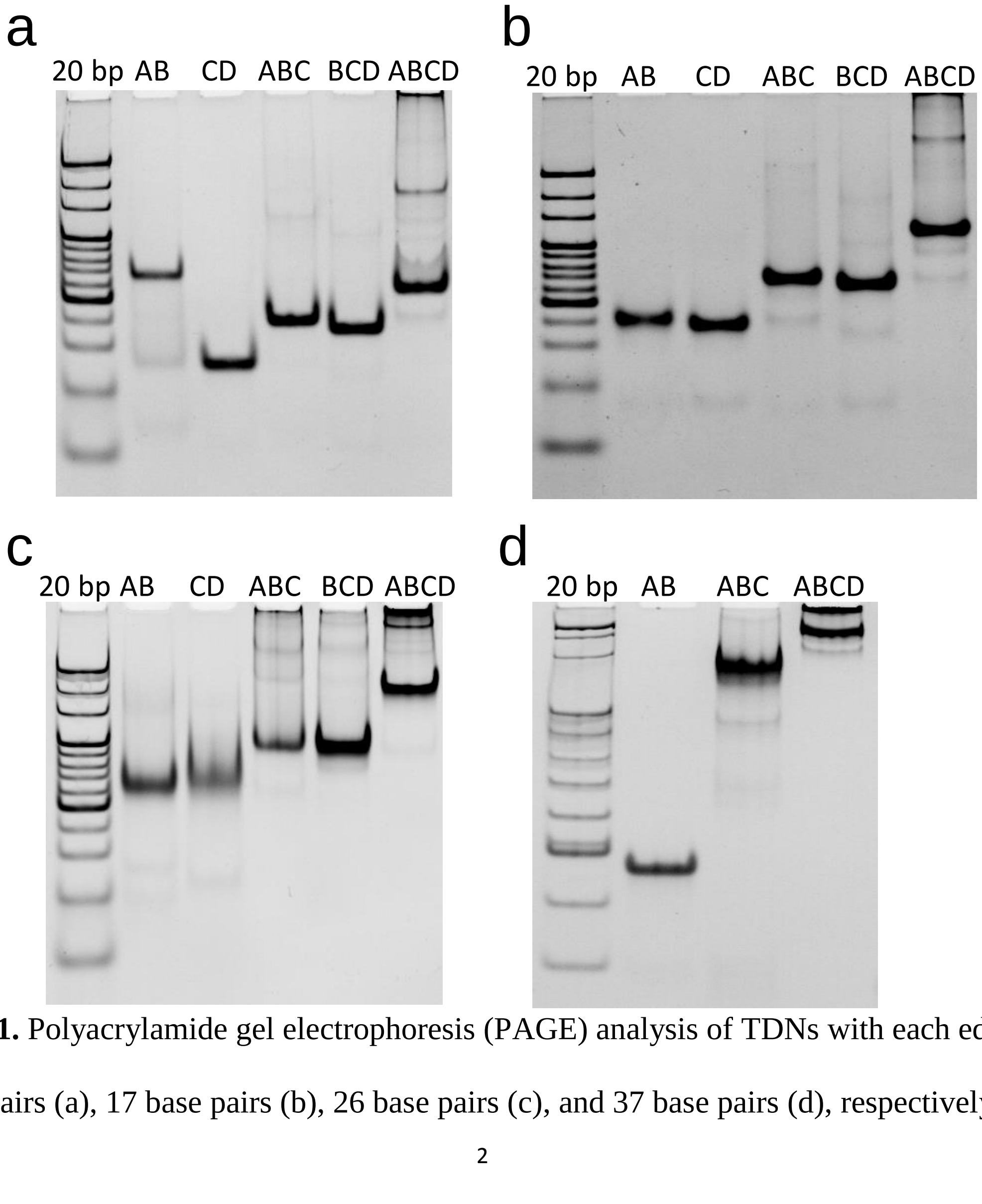

Figure S1. Polyacrylamide gel electrophoresis (PAGE) analysis of TDNs with each edge of 13 base pairs (a), 17 base pairs (b), 26 base pairs (c), and 37 base pairs (d), respectively.

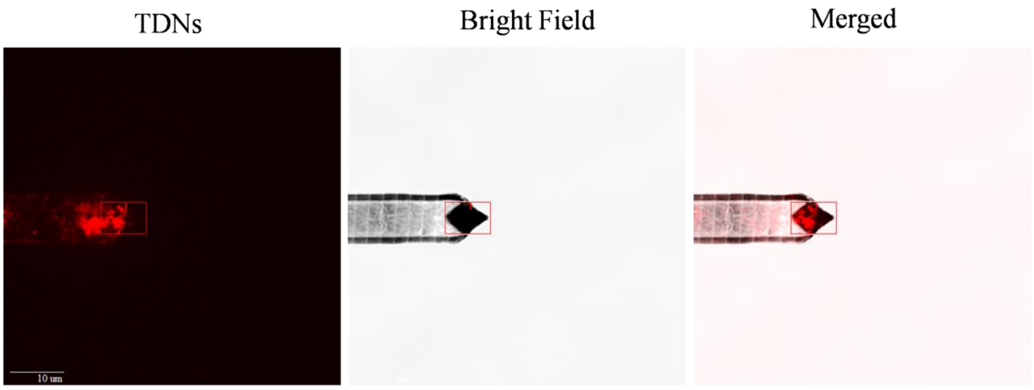

Figure S2. The fluorescence images of AFM tip with two-color confocal microscopy. The red particles represent TDN-37. Scale bar: 10 µm.

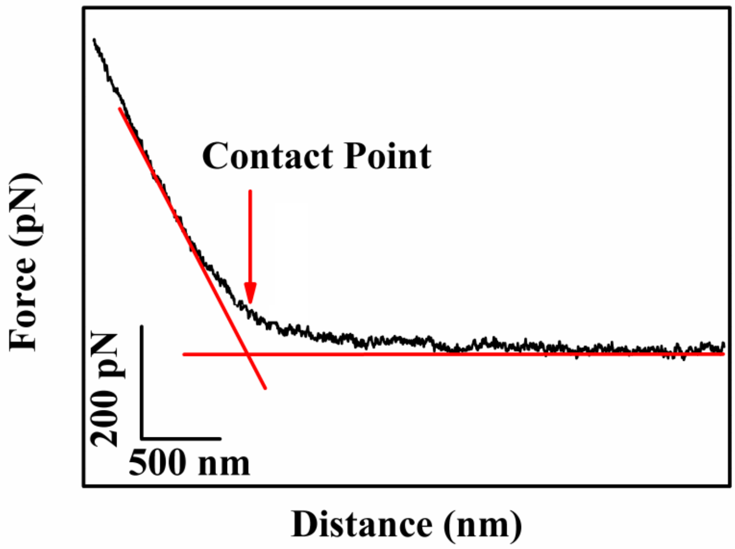

Figure S3. Engaging the TDN-13 modified AFM tip to the cell surface and finding the contact point at which the AFM tip lightly contacts the cell membrane, as shown the red arrow. The contact point is the intersection of flat part and slope (red lines) in the force-distance curve.

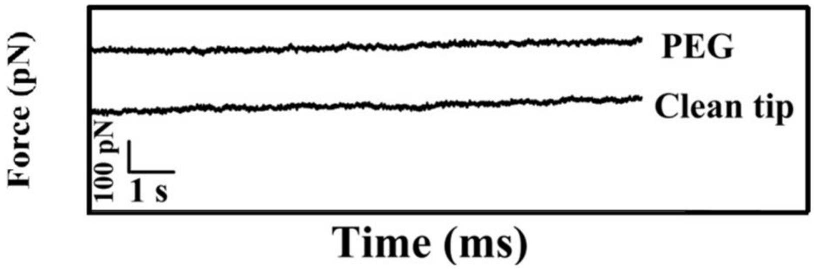

Figure S4. Control experiments, the typical force tracing curves for clean AFM tip and only PEG modified AFM tip on the HeLa cells.

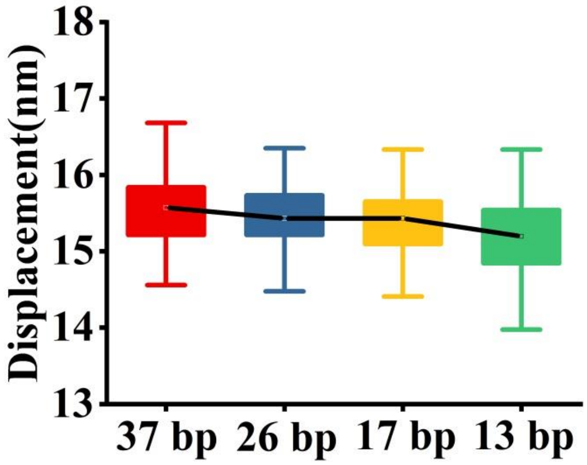

Figure S5. The displacement of TDNs with different size (TDN-13, TDN-17, TDN-26, and TDN-37) during trans-membrane transporting.

Details for simulation method and model

Dissipative particle dynamics (DPD) method is a coarse-grained simulation technique, which can correctly account for the hydrodynamic interactions by considering water molecules explicitly.[2] In DPD simulations, a cluster of atoms is represented by a bead whose dynamics is governed by Newton's equation of motion $dv_{ij}/dt = F_{ij}/m$. Beads i and j interact through simple pairwise force consisting of a conservative force (F_{ij}^{c}), a dissipative force (F_{ij}^{D}), and a random force (F_{ij}^{R}). Therefore, the total force is applied on each bead i, since the bead j is given as a sum of these three terms:

$$F_{ij} = (F_{ij}^{C}) + (F_{ij}^{D}) + (F_{ij}^{R})$$

(1)

The conservative force is $F_{ij}^{C} = a_{ij}\omega\left(a_{ij}\right)n_{ij}$, where a_{ij} is the repulsive strength, $r_{ij} = r_i - r_j$ (r_i being the position of bead i), $r_{ij} = |r_{ij}|$ and $n_{ij} = r_{ij}/\gamma_{ij}$ is the unit vector. Dissipative force $F_{ij}^{D} = -\gamma\omega^2(r_{ij})(n_{ij}\, v_{ij})\, n_{ij}$ with $v_{ij} = v_i - v_j$ (v_i being the velocity of bead i). Random force $F_{ij}^{R} = \sigma\omega(r_{ij})\theta_{ij}(\Delta t)^{-1/2}\, n_{ij}$, $\theta_{ij}(t)$ is a random number with zero mean and unit variance, Δt is the time step. The function $\omega(r_{ij})$ is defined as

$$\omega(r_{ij}) = \begin{cases} 1 - \dfrac{r_{ij}}{r_c}, & r_{ij} < r_c \\ 0, & r_{ij} > r_c \end{cases}$$

(2)

Where r_c is the cutoff radius. The parameters r and σ are related to each other as $\sigma^2 = 2rk_BT$, where k_B is Boltzmann constant and T is the absolute temperature. Moreover, in order to mimic the receptor-ligand interaction, we use a modified LJ potential[3]:

$$U^{LJ}(r_{ij}) = 4\varepsilon\left[(\frac{\sigma}{r_{ij}})^{12} - (\frac{\sigma}{r_{ij}})^{6}\right] + 0.22\varepsilon, \quad r_{ij} < r_{cut}$$

(3)

Where $\sigma = 0.624r_c$, and ε is used to identify the strength of the receptor-ligand interaction. Furthermore, we use a harmonic bond $U_{bond} = K_{bond}(1 - \frac{r_{ij}}{l_0})^2$. We also use a three-body bond angle potential $U_{angle} = k_{angle}(1 - \cos(\varphi - \varphi_0))$ to depict the rigidity of lipid tails and the ligands. In the simulation, the cell membrane is formed by a collection of lipids and receptors as a stable bilayer structure.[3] Similar to the lipid model developed by P. B. Sunil Kumar *et.al.*,[4] the lipid/receptor molecule is represented by 4 spherical beads, as shown in Figure S6. The head of lipid molecule is formed by 1 hydrophilic bead (H), while the tail is formed by a chain with 3 hydrophobic beads (T). To represent the hydrophilic/hydrophobic property of the

head and tail beads in the lipid molecule, the repulsive interaction parameters for the same type of beads are a_{ij} = 25, and other interaction parameters are listed in Table S2. The neighboring beads i and j in the lipid are connected together by a simple harmonic spring with a spring constant of $k_{bond} = 100 \times \varepsilon/r_c$ and an equilibrium bond length of $l_0 = 0.45 \times r_c$. The force constraining the bond angle is described by an equilibrium angle of $\varphi_0 = \pi$ and a bond bending force constant of $k_{angle} = 10 \times \varepsilon$ for three consecutive tail beads. The TDN is formed by beads arranged as a tetrahedron and constrained to move as a rigid body during the simulations.

In the simulation, the units are all normalized. The unit of length is taken to be the cutoff radius r_c, the unit of the mass is that of beads, and the unit of energy is taken to be k_BT. Other quantities are given in terms of these basic units. In terms of normalized units, we use the standard values for σ = 3.0 and r = 4.5. The time step Δt = 0.01 τ and $\tau = \sqrt{mr_c^2 / k_BT}$ is the unit of the time. The simulation box is a cube of size $80r_c \times 80r_c \times 60r_c$ with periodic boundary condition applied three directions. There are total 1152000 beads in the simulation box to keep the particle density is close to 3.0. To avoid the distribution of the nanoplate to the equilibrium state of a pure membrane, a long time (~200 ns) equilibrium simulation was preformed when the DNA was placed near the membrane surface.

During the simulation of the NP/NR-membrane interaction, to maintain the zero lateral tension, the lipid number per area (ρ_{LNPA}^{BR}) at the boundary region is adjusted to keep a constant by stretching or compressing the simulation box. In this case, the central square region of the membrane ($60r_c \times 60r_c$) is surrounded by a reservoir of lipids with a width of $5r_c$ along the x and y directions. In practice, we performed one stretching/compressing move every 1200 time steps in order to leave enough time to propagate the membrane tension to the whole membrane. Since the membrane tension is directly related to (ρ_{LNPA}^{BR}), when the area per lipid is

about $0.7r_c^2$ (corresponding to $(\rho_{LNPA}^{BR}) = 1.43$), the surface tension of the lipid bilayer becomes zero.[5]

Table S2. Values of interaction parameter a_{ij} between two beads in DPD simulations.

a_{ij}	H_{lipid}	T_{lipid}	$H_{receptor}$	$T_{receptor}$	DNA	W
H_{lipid}	25	200	25	200	25	25
T_{lipid}		25	200	25	200	200
$H_{receptor}$			25	200	25	25
$T_{receptor}$				25	200	200
DNA					25	25
W						25

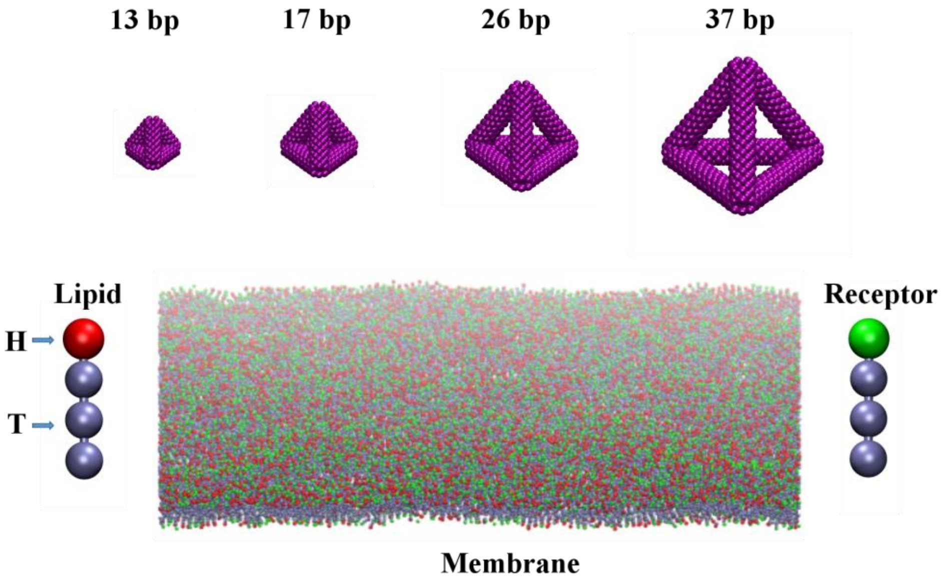

Figure S6. Schematic illustration of the model in simulation. Purple beads formed tetrahedron represents the DNA, red bead represents lipid head, iceblue beads represent the tail of lipid and receptor, green bead represents the head of the receptor. The conformation of the membrane and TDNs with different size are shown after equilibration in water. The membrane is consisted of 9100 lipids and 9100 receptors. For clarity, the solvent (H_2O) molecules are made invisible.

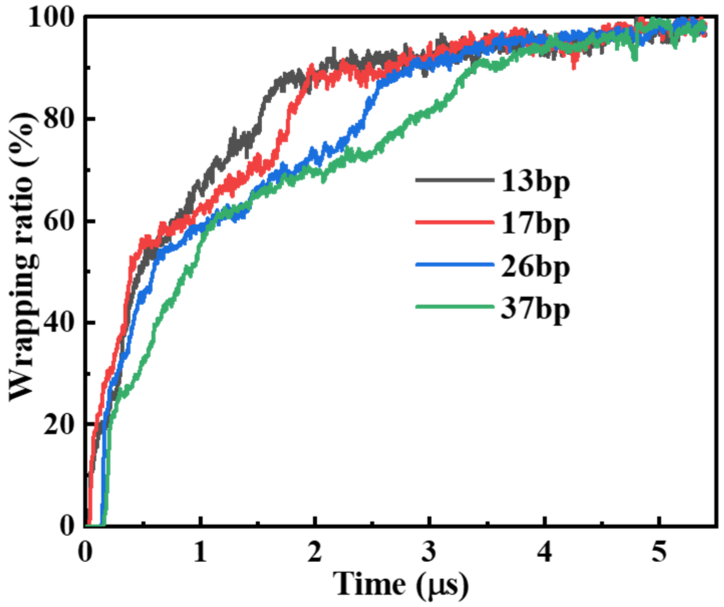

Figure S7. The percentage of wrapping TDNs as time going. The distance values for determining the ligand-receptor binding is selected within a distance of 0.7 r_c.

References

[1] H. Pei, L. Liang, G. Yao, J. Li, Q. Huang, C. Fan, *Angew. Chem.-Int. Edit.* **2012**, *51*, 9020.
[2] R. D. Groot, P. B. Warren, *J. Chem. Phys.* **1997**, *107*, 4423.
[3] M. Laradji, P. B. Kumar, *Phys. Rev. E* **2006**, *73*, 040901.
[4] M. Laradji, P. B. Sunil Kumar, *Phys. Rev. Lett.* **2004**, *93*, 198105.
[5] F. L. Tian, T. T. Yue, W. Dong, X. Yi, X. R. Zhang, *Phys. Chem. Chem. Phys.* **2018**, *20*, 3474.
